# User involvement in the implementation of clinical guidelines for common mental health disorders: a review and compilation of strategies and resources

**DOI:** 10.1186/s12961-016-0135-y

**Published:** 2016-08-09

**Authors:** Eliana M. Moreno, Juan Antonio Moriana

**Affiliations:** 1 Department of Psychology, University of Cordoba, Calle San Alberto Magno S/N, 14071 Cordoba, Spain; 2 Maimonides Institute for Research in Biomedicine of Cordoba (IMIBIC), Reina Sofia University Hospital, Avda Menéndez Pidal s/n, 14004 Cordoba, Spain

**Keywords:** Mental health, Implementation, Service users, Health resources, Clinical practice guidelines

## Abstract

**Background:**

There is now broad consensus regarding the importance of involving users in the process of implementing guidelines. Few studies, however, have addressed this issue, let alone the implementation of guidelines for common mental health disorders. The aim of this study is to compile and describe implementation strategies and resources related to common clinical mental health disorders targeted at service users.

**Methods:**

The literature was reviewed and resources for the implementation of clinical guidelines were compiled using the PRISMA model. A mixed qualitative and quantitative analysis was performed based on a series of categories developed ad hoc.

**Results:**

A total of 263 items were included in the preliminary analysis and 64 implementation resources aimed at users were analysed in depth. A wide variety of types, sources and formats were identified, including guides (40%), websites (29%), videos and leaflets, as well as instruments for the implementation of strategies regarding information and education (64%), self-care, or users’ assessment of service quality.

**Conclusions:**

The results reveal the need to establish clear criteria for assessing the quality of implementation materials in general and standardising systems to classify user-targeted strategies. The compilation and description of key elements of strategies and resources for users can be of interest in designing materials and specific actions for this target audience, as well as improving the implementation of clinical guidelines.

## Background

The introduction of new models in the mental healthcare field, such as the person-centred approach [1–3] or the recovery model [4–7], has led to substantial reforms in the care of various disorders. Among other aspects, this situation has resulted in the development of clinical guidelines and the implementation of evidence-based treatments in public health systems. Moreover, the roles of the various bodies involved have changed, with users beginning to take a greater role [8, 9]. Currently, there is widespread consensus regarding the benefits of involving users, families and caregivers in the planning and design of services and health policies [8, 10, 11]. With this aim, various types of interventions have been proposed, such as providing information and education, fomenting collaborative decision making, and promoting self-care [2, 12, 13]. One of the most common actions has been to enhance user involvement in the process of developing clinical practice guidelines [14–16], which also constitutes a quality criterion for such guidelines [17, 18]. One of the leading organisations working in this line is the National Institute for Health and Care Excellence (NICE), which involves patients and those affected in the consultation process and design of the guidelines it develops, as well as the processes to implement them [19, 20].

A key element for the success of evidence-based guidelines is to engage the various stakeholders involved. To this end, it is necessary to motivate and involve managers, professionals and users in implementation plans [21, 22]. In this regard, it has been shown that strategies targeting specific groups and materials designed specifically for different types of audiences are most effective [2, 23]. Examples of user-targeted interventions include the use of adapted versions of guidelines, the awarding of prizes, increased or reduced patient fees, co-payment, and mechanisms to obtain feedback or gather/channel complaints and suggestions. Although there is abundant literature on implementation strategies in general, few studies have focused on service users [24, 25], while even fewer studies have been conducted on the implementation of guidelines for common mental health disorders. These studies include disorders such as anxiety or depression, which have a moderate level of severity but are highly prevalent in the adult population worldwide [26]. For these reasons, it is important to deepen the understanding of more specific and less widespread strategies.

### Objectives

The aim of this study was to compile and describe the strategies and resources used to implement clinical guidelines for common mental health disorders targeted at service users.

## Method

We reviewed and compiled resources designed or selected by NICE for the implementation of clinical guidelines for common mental health disorders in adults to further the analysis of user-centred materials and strategies. In order to improve the clarity and transparency of the review process, we followed the criteria and key recommendations of the PRISMA Statement [27, 28].

### Search strategy

The primary source we consulted was the official website of NICE. Due to the significant amount of information the website provides, the resources were located by means of two strategies. The first strategy was to identify implementation materials contained in reference guides on common mental health disorders that are currently available [29–35]. In each of these guides, we consulted the ‘Tools and resources’ section, which contains subsections providing access to documents, materials and resources. The second strategy involved identifying other resources designed to enhance user involvement in the implementation process. To do so, we consulted the ‘Service delivery, organization and staffing’ section, where we located related materials under the ‘Patient and service user care’ tab. Once all the items were located, duplications were eliminated and eligibility criteria were applied.

### Eligibility criteria

The selection process was conducted in two phases. In the first phase, we selected the materials required to perform a comprehensive preliminary analysis according to the following inclusion criteria: (1) all resources targeted at managers, professionals, users and the general public that were directly or indirectly related with the implementation of clinical guidelines for common mental health disorders in adults; (2) various types of resources, including clinical guidelines, scientific articles, reports, manuals, tools, online resources, audio-visual materials, and educational materials; (3) considering the years 2005–2015; and (4) only published materials. We excluded: (1) items not related to common mental health disorders; (2) items unrelated to implementation; (3) items solely related to legislative issues or regulations; (4) items targeting children and/or youth and the over-65 population; and (5) developing materials or guidelines.

**Fig. 1:**
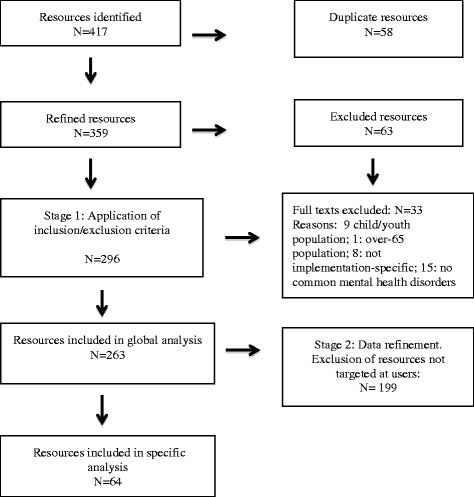
PRISMA flow diagram

In the second phase, we refined our search to select only those items specifically designed for or targeted at service users, family members and caregivers. Items intended exclusively for professionals, managers or other audiences were excluded. The process to identify and select the items using the PRISMA model is shown in Fig. 1.

### Data collection

Step 1. All the items identified in the search were entered into a database designed to sort the following general information: name, year of publication, language, pages/extension, and references.

Step 2. After reviewing different models and taxonomies to classify implementation actions and strategies, an ad hoc template with a series of specific categories and subcategories was developed. Elements from the Cochrane Review Group’s Effective Practice and Organisation of Care (EPOC) protocol [36, 37] and other relevant studies were used as a reference [25, 38–41]. All the resources that met the inclusion criteria were analysed in full based on these categories. A list and description of the categories are provided in Table 1.

**Table 1:** Categories for the analysis of user-targeted implementation resources

Category/Description	Subcategories
*Type of resource* General characteristics or structure of presentation	Guide/Manual; Book/Document; Leaflet; Course; Website; Instrument (scale/questionnaire/self-report/other); Video/Podcast; Other
*Format* Format or medium in which the resource is available	Interpersonal (face-to-face/by telephone); PDF/Word/Excel; Audio/visual; Website/online; other
*Source* Organisation, institution, group developing the resource	Local clinicians/local expert body; National professional expert body; National government expert body; International professional expert body/international government expert body; Non-governmental organisations/foundations/national associations; Agency/national company
*Language*	English; Welsh; Other
*Target audience* Specific target population for action	Professionals/managers; Users/patients; Users/family members/caregivers
*Type of implementation strategy* Intervention(s) or action(s) aimed at facilitating the implementation of recommended guidelines	I *Develop and distribute educational materials*: Design and deliver manuals, tools and other support materials to aid in understanding guidelines or recommendations II *Prepare patients/consumers to be active participants*: Strategies/actions to encourage users to be more actively involved in their own healthcare III *Develop and implement tools and systems for quality monitoring*: Actions designed to implement systems and procedures to evaluate the quality of care; promote the use of protocols, standards, and measures to evaluate service performance and determine to what degree practices comply with recommended guidelines
*Specific objective* Specific objective or purpose of the resource	Related to Strategy I: Provide accessible and easy-to-understand information/education on various issues: a) Content of guidelines/recommendations; b) evidence-based recommendations; c) characteristics of the disorder/condition (symptoms, evolution, prognosis, etc.); d) information on the intervention (treatment(s), procedure(s), options, features, pros and cons, etc.); e) information on social service care (available resources, aid, services) Related to Strategy II: a) Self-care: Materials/actions to enhance patients’ involvement in their own healthcare or the self-management of various health conditions. This includes, for example: a.1) training programmes (relaxation techniques, problem-solving skills, etc.); a.2) materials for self-reporting and self-monitoring of symptoms; a.3) material on symptom management or special situations (e.g. crises, relapses) b) Self-help: Resources/actions to encourage support and help among peers or guided by professionals b.1) access to self-help books/guides; b.2); access to peer groups; b.3) provide interactive support-care by telephone or information technologies (chats, e-mails, blogs); b.4) mixed/unspecified Related to Strategy III: a) Define quality standards (users’ version): resources designed to promote users’ capacity to assess/request services, quality care, and treatment consistent with guidelines and recommendations

Step 3. To facilitate the data collection and analysis, a checklist system was also developed. Doubts regarding the categorisation of items were resolved in consensus with an expert in the field of health management who was not involved in the research.

### Analysis strategies

The data analysis was carried out in two stages. In the first stage, we performed a general quantitative analysis to measure the frequency and percentages of all the resources included in the first selection phase. In the second stage, following the refinement of the data, we performed a mixed qualitative and quantitative descriptive analysis of the data drawn from resources targeted only at users. Measures of frequency and percentages related to the previously designed categories were then obtained.

## Results

### Selection process

Different resources to implement seven clinical practice guidelines on common mental health disorders, including anxiety and panic disorder, post-traumatic stress, social anxiety, obsessive-compulsive and body dysmorphic disorders, and depression in adults, were reviewed [29–35]. These guides contain evidence-based recommendations for appropriate treatment at the psychological, psycho-educational or pharmacological level.

A total of 417 resources were initially identified, which resulted in 359 resources after eliminating duplications. Of these, 63 were excluded based on the title and abstract. Following the first refinement process, 33 documents that did not meet the inclusion criteria applied in Phase 1 were eliminated. These comprised documents that were (1) targeted at children and youth or the over-65 population; (2) not directly or indirectly related to implementation; and (3) not associated with common mental health disorders. This first phase yielded 263 resources, which were used to conduct a preliminary analysis of all the available materials targeted at different audiences.

In the second refinement phase, 64 resources targeted specifically at service users were selected and analysed in depth. The selection process following the PRISMA approach is shown in Fig. 1.

We performed a global analysis of the 263 resources to calculate the proportion of resources targeted at each type of audience. We found that most of the resources are designed for use by professionals (40%) and managers (35%), whereas only 24% are specifically for users.

### General characteristics of the user-targeted resources

#### Types and contents of resources

The most frequent type of resources are guides or manuals, which account for almost 40% of all resources. These included (1) adapted versions of each of the clinical guides on common mental health disorders (anxiety and depression), which provide a summary of evidence-based recommendations and available treatments in an easy-to-understand language, in addition to information suited to the needs of users and the general public; (2) quality standards, which explain the type of intervention(s) and care that patients should receive according to pre-established quality criteria; and (3) a self-help guide, which contains links to most of the resources analysed, as described below.

The second most frequent type of resource are links to websites of several specialised agencies or foundations (29.7%), which contain specific information and materials to promote self-care among users. Videos and podcasts account for 12.5% of the available resources. These materials provide visual or audio information that primarily narrate the experiences of individuals with the disorder and those undergoing treatment. They are also used to teach different types of skills (social skills, relaxation techniques, etc.).

Other materials, such as books, leaflets, tools (e.g. self-assessment/monitoring of symptoms), as well as training courses for patients, family members and caregivers, were also found. The number and percentage of resources grouped into each of the general categories are summarised in Table 2.

**Table 2:** Summary of general characteristics of resources included in the study

Category	Subcategories	N (%)
Type of resource	Guide/Manual	25 (39%)
	Book/Document	4 (6.2%)
	Leaflet	4 (6.2%)
	Course	1 (1.5%)
	Website	19 (29.7%)
	Instrument (scale/questionnaire/self-reporting/other)	2 (3.1%)
	Video/Podcast	8 (12.5%)
	Other: Self-help line	1 (1.5%)
Format	Interpersonal (face-to-face/by telephone)	1 (1.5%)
	PDF/Word/Excel	32 (50%)
	Audio/visual	8 (12.5%)
	Web/online	23 (36%)
Source	Local clinicians	1 (1.6%)
	National professional expert body	7 (10.9%)
	National government expert body	30 (46.9%)
	International professional expert body	1 (1.6%)
	Non-governmental organisations/foundations/national associations	18 (28.1%)
	Agency/national company	7 (10.9%)
Language	English	54 (84.3%)
	Welsh	10 (15.6%)
	Other (*Translation of online content in different languages)	19 (29.7%)
Target audience	Users/patients	55 (85.9%)
	Users/family members and caregivers	9 (14%)

#### Format, source and target audience

Most of the above materials are available in downloadable PDF format (50%) or in the form of online support (36%).

The main source is the NICE, which designs and disseminates almost 47% of the implementation resources, followed by national foundations and non-governmental organisations (i.e. the Mental Health Foundation, Anxiety UK or Mind), which develop 28.1% of the materials, and national professional bodies (i.e. the Royal College of Psychiatrists), which develop almost 11% of all resources. These materials are targeted mainly at patients and individuals with common mental health disorders (85.9%), particularly some type of anxiety, while 14% are more specifically aimed at supporting family members and caregivers.

#### Specific characteristics: strategies and objectives

The items analysed were grouped into three key implementation strategies (Table 3). The most widely used strategy is the development and dissemination of educational materials (64%), which mainly provide easy-to-understand information on the content of the guides, the characteristics of the disorder, and the intervention. The second most common strategy is to prepare users to take a more active and participatory role in their own healthcare. This group includes materials whose main objective is to promote self-help (15.6%) and self-care (11%), such as guidelines for developing relaxation or problem-solving skills, access to peer groups and self-help blogs, self-reporting instruments and tasks to perform at home, and advice for managing symptoms, crises or preventing relapses. The third strategy relates to the development of tools and systems that enable quality monitoring. Of these, 9.4% of the materials are guides with quality indicators in versions adapted specifically for users.

**Table 3:** Summary of implementation strategies and specific objectives of the resources included in the study

Type of strategy, N (%)	Specific objective, N (%)	Content/specific action	N (%)
Develop and distribute educational materials, 41 (64%)	Information/education, 41 (64%)	Content of guidelines/recommendations	14 (21.9%)
		Characteristics of disorder/condition (symptoms, evolution, prognosis, etc.)	11 (17.2%)
		Information on intervention (treatment(s), procedure(s), options, features, pros and cons, etc.)	13 (20.3%)
		Information on social services/care (available resources, aid, services)	3 (4.7%)
Prepare patients/users to be active participants, 17 (26.6%)	Self-care, 7 (11%)	Training programmes/material (relaxation techniques, problem-solving skills, etc.)	3 (4.7%)
		Material for self-reporting or self-monitoring symptoms	2 (3.1%)
		Material/advice on symptom management or special situations (e.g. crises, relapses)	2 (3.1%)
	Self-help, 10 (15.6%)	Material to support/access peer groups	4 (6.2%)
		Access to interactive support care by telephone or information technologies (chats, mails, blogs)	1 (1.6%)
		Mixed, unspecified	5 (7.8%)
Develop and implement tools and systems for quality monitoring, 6 (9.4%)	Assessment of services and quality of care, 6 (9.4%)	Quality standards	6 (9.4%)

## Discussion

The analysis of the most important results revealed three key points. First, it should be noted that after reviewing the literature on the topic and examining the main taxonomies proposed, we detected a significant heterogeneity in the classification of user-targeted strategies and interventions and a lack of specificity in some descriptions. For this reason, we believe that it is necessary to establish a common criteria for defining actions aimed at users and users’ role in the complex process of implementing guidelines.

The second point refers to the main findings regarding the general characteristics of the resources. According to several authors, adapted versions of clinical guidelines are one of the most widely disseminated types of material on implementation aimed at users [15, 16, 38]. However, we found that these materials account for only a small proportion of all resources in contrast to other types of guides, manuals or tools. In this study, people with a problem (patients or those affected) are considered as service users. However, in a more inclusive perspective, the user concept also covers people that provide practical or emotional support to someone with a mental health problem, like family members or caregivers. In this line, all the resources that we located are designed for a specific target audience (patients or those affected, family members, caregivers or even the work setting). Most of them are targeted at patients and includes resources, such as self-help guides, informative guides or guides on quality assessment, as well as a wider variety of general resources, including specialised websites, videos, audio recordings, leaflets, books, self-assessment tools or courses. Resources specifically targeted at family members or caregivers includes guides, books or specific websites with information on how to help or how to cope with the pressure of being a carer.

This reveals that implementation materials are available in a wide range of types, contents and formats whose design is an important aspect that must be taken into consideration. These resources use easy-to-understand language and contain simple content adapted to the specific needs of users. Moreover, they have specific objectives and are usually rather short in length. In addition, certain formats, such as videos or podcasts, often employ members of the community to transmit the information (e.g. other patients or members of patient associations) in order to help users understand or assimilate the messages. In this line, several studies have reported that complex actions covering different objectives, levels and target audiences, together with the use of materials designed specifically to take into account the characteristics of the population they serve, increase the effectiveness of interventions [24, 40, 42, 43]. Therefore, the points outlined above could be considered key elements for the successful development and dissemination of implementation resources.

With regard to who designs and develops these tools, we found that, although the NICE produces nearly half of the materials we located, bodies external to the organisation that develops the guides are also involved. This could be interpreted as the use of implementation strategies to forge alliances or partnerships with professional groups and non-governmental organisations [41] in order to produce or distribute materials to assist in the implementation of the recommendations. This indicates that NICE plays an important role in producing such materials but also in the selection of other existing ones. From a more global perspective, it is important to note that public health agencies assume responsibility for the development of specific and well-designed materials to ensure that the contents and formats are appropriate. However, it is also important that they classify and assess the quality of the resources developed and disseminated by other entities, groups or companies for two reasons. First, due to the enormous wealth of available information and materials, especially those related to self-help, and second, because users often do not have the adequate tools to assess the suitability of such resources or to detect biased information. This highlights the need to define clear criteria for assessing the quality of the resources. Moreover, providing access to materials that have already been ‘filtered’ and organised in an appropriate manner can ensure greater user safety and the effectiveness of the implementation strategies.

The last point relates to the implementation strategies used, which can be summarised in three key issues. The first refers to the strategy of providing information and education about different topics (the disorder, symptoms, evolution, treatment options, etc.). The second relates to actions to enhance users’ involvement in their own healthcare. In this regard, self-help and self-care resources increase patients’, families’ and caregivers’ feelings of control and self-efficacy regarding their own healthcare by providing not only support resources, but also by improving coping and management skills in diverse areas. Some studies have highlighted that users with mental health problems manifest the need for more information and to strengthen their involvement in recovering their health [44–46]. Both types of strategies help to improve health literacy. These actions enhance users’ awareness of their state or condition and can help increase engagement in the decision-making process [47, 48]. Thus, it is recognised that, in practice, shared decision-making is infrequent due to, among other things, the lack of tools to involve patients in the process [2]. It is therefore important to have tools which provide patients the resources they require to assume a more active role in the decision-making process. This last element is crucial to the person-centred approach and a fundamental objective of clinical practice guidelines [49].

The third implementation strategy we detected is users’ involvement in assessing the quality of services, which is one of the most current although infrequently mentioned strategies in the literature [7]. The aim of the guidelines on quality standards [50, 51] is not simply to gain feedback from patients or obtain clinical outcomes, but to address broader issues such as the use of informed consent and shared decision-making. Such guidelines may empower users by providing them a tool to compare and contrast the degree of consistency between interventions, the actual treatment they receive, and recommendations. In this manner, users may judge the care they receive based on clear criteria and request interventions that comply with the guidelines.

### Limitations

Despite NICE probably having one of the most extensive lists of resources, one of the main limitations of this study is that a single source of information (the NICE website) was used. In future research, it would be of interest to broaden the search strategy in order to identify and analyse other sources of information, as well as types of strategies and resources such as those of a mass, interactive or face-to-face nature.

## Conclusions

In this study, we have presented a compilation and qualitative synthesis of the characteristics of various user-targeted implementation resources that can contribute to the development of new materials and strategies for the implementation of clinical guidelines. Although we have focused on the implementation of common guidelines for mental health disorders, some of the key results can be applied to other settings and disorders.

The analysis reveals that the way in which the resources are designed plays a very significant role. Features related to both content and format can not only improve the implementation of clinical recommendations, but also facilitate shared decision-making processes and person-centred care.

We have emphasised the important role users play in the implementation process, although little attention has been paid to this topic in the literature. We have also highlighted the importance of establishing common criteria for assessing resources and materials and improving the description of user-focused implementation strategies.
